# Use of a Shoulder-Mounted Wearable Sensor Prototype Designed to Detect Opioid-Related Overdose: A Qualitative User Experience Study

**DOI:** 10.2196/73566

**Published:** 2025-07-28

**Authors:** Alexis M Roth, Ally K D'Angelo, David Gordon, Benjamin Cocchiaro, Anush Lingamoorthy, Rose Laurano, Matthew Salzman, Jacob S Brenner, Cameron Baston

**Affiliations:** 1 Department of Community Health and Prevention, Dornsife School of Public Health, Drexel University, 3215 Market Street, Philadelphia, PA, 19104, United States, 1 2154330457; 2 Sidney Kimmel Medical College, Thomas Jefferson University, Philadelphia, PA, United States; 3 Dornsife School of Public Health, Drexel University, Philadelphia, PA, United States; 4 Department of Electrical and Computer Engineering, College of Engineering, Drexel University, Philadelphia, PA, United States; 5 Department of Emergency Medicine, Cooper Medical School of Rowan University, Philadelphia, PA, United States; 6 Department of Medicine, Division of Pulmonary, Allergy and Critical Care, Perelman School of Medicine, University of Pennsylvania, Philadelphia, PA, United States

**Keywords:** overdose, wearable device, prevention, biosensor, acceptability

## Abstract

**Background:**

For over a decade, drug overdose has been the leading cause of injury and accidental death in the United States. Most fatal overdoses involve opioids and occur during solitary drug use events when no one is available to initiate lifesaving responses (eg, naloxone). While there is a growing interest in devices providing early overdose detection and automated responses, little research has engaged end users in a device design process.

**Objective:**

This study aimed to describe user experience, perceived harms and benefits, and the acceptability of a shoulder-mounted wearable sensor among people who inject drugs who wore a device prototype for 7 days and to explore real-time responses that could be incorporated into a next-generation sensor.

**Methods:**

Individuals aged ≥18 years reporting past week injection drug use were recruited from a walk-in substance use disorder clinic in Camden, New Jersey. Participants completed a brief survey assessing sociodemographics and recent drug use and were assigned a sensor prototype that they were asked to wear near-continuously for 7 days. At endline, they completed semistructured interviews exploring acceptability, usability, and form and function preferences for next-generation devices with integrated automated response options. Structured field notes and transcripts were analyzed to identify key themes and design considerations.

**Results:**

Participants (n=23) had a median age of 41 years. Most were primarily non-Hispanic White (65%) males (61%), experiencing homelessness (57%) who reported daily injection drug use (74%) within 3 months and receipt of opioid use disorder medication within a month (74%). A total of 16 people completed an exit interview. They found the concept of a shoulder-mounted overdose detection device acceptable and unanimously endorsed the need for long-lasting discreet devices. They emphasized the importance of having multiple response options that wearers could tailor to their individual circumstances and preferences, noting some might prefer an automatic call to emergency services, while others might prefer to alert a peer. Participants expressed a preference for solutions that alert first responders over automated biomedical solutions, such as naloxone injection, because of concerns about device error (eg, false positives) leading to precipitated withdrawal.

**Conclusions:**

After wearing a shoulder-mounted prototype, this small group of participants found the idea acceptable and provided feedback to improve usability and design. Data suggest that a variety of devices with differing functions, sizes, and capacities will be needed to meet user needs and increase the likelihood of adoption once devices come to market.

## Introduction

### Background

Of the more than 100,000 fatal overdoses in the United States in 2023, most (69%) were related to synthetic opioids, such as illicitly manufactured fentanyl [1,2]. Opioid overdoses primarily impair the brain’s respiratory centers, leading rapidly to hypoxia—a dire neurological emergency that necessitates immediate intervention [3]. Emergency responders have multiple ways to address overdose, but the primary pharmacological treatment is naloxone [4]. Despite its lifesaving potential, naloxone’s effectiveness is critically hampered by delays in overdose recognition and response [5]. Overdose events often go unrecognized when bystanders are present, and more than half of overdoses occur when someone uses drugs alone and there is no one there to administer naloxone [2,6,7]. The window to prevent irreversible brain damage or death once oxygen levels become low is perilously short (less than 5 minutes), with hypoxic injury beginning within minutes after an overdose [3,8]. Thus, we urgently need solutions that quickly identify an overdose and facilitate immediate evidence-based emergency response like rescue breaths to artificially ventilate the lungs or naloxone administration when appropriate.

Further complicating overdose response is the increasing presence of veterinary sedatives like xylazine and medetomidine as adulterants in the illicit supply of synthetic opioids like fentanyl [9-11]. In Philadelphia, for example, medetomidine has largely replaced xylazine following increased xylazine supply controls implemented in the state in 2024 [12]. Although naloxone reverses opioid-related overdoses, it does not address the novel respiratory depression and cardiac effects produced by the synergistic effects of fentanyl and nonopioid sedatives [12-16]. Public education surrounding naloxone focuses on opioid reversal with limited discussion of alternative interventions like rescue breathing or bag-valve-mask ventilation for sedatives not reversed by naloxone. This gap has created an unfortunate situation wherein the focus on “naloxone first” results in repeated and ineffective doses of naloxone, without directly addressing the respiratory suppression causing mortality risk [17]. Moreover, the impact of receiving unneeded doses of naloxone on people who use opioids is profound, with individuals experiencing severe and prolonged symptoms of opioid withdrawal such as pain, agitation, anxiety, and vomiting [18,19].

Wearable biosensors with the capacity for continuous physiological monitoring of key indicators of overdose like respiratory rate, heart rate, oxygen saturation, and movement have been proposed as a novel approach to facilitate reliable and timely detection of and response to opioid overdose [20,21]. Biosensors could also integrate necessary components for computational modeling and interpretation of the relevant physiological parameters to determine the severity of an overdose and to deliver interventions customized to the individual’s physiological situation. Thus, biosensors represent an innovative approach to dramatically reduce overdose response time. By bringing medical resources to the patient before physiological damage has begun to occur, the clinical outcomes of an overdose event may be improved.

Over the past decade, several wearable technologies have been proposed or developed to address opioid overdose. The Masimo Halo is the first and only Food and Drug Administration (FDA)–approved device for detecting opioid-induced hypoxemia using pulse oximetry [22]. It uses a single-use fingertip pulse oximeter, which sends data to a base station wirelessly using Bluetooth, and from there to a smartphone for classification and alert. The optical sensor has a battery life of less than 4 days and costs US $90 to replace. The overall cost of the Halo unit is around US $250. That device allows for remote monitoring for housed individuals or individuals in congregant housing who have sufficient connectivity to use its multiple components while indoors. However, it is not useful for patients without stable housing, smartphones, and the ability to frequently charge a device. In addition, the fingertip and watch configuration was ranked as less likely to be worn by people who use opioids [23].

Other research-based solutions, such as smartphone-based monitors [24] or wearable respiratory rate monitors, use either a single biomarker approach, leading to a much higher risk of false positive alarms than a multiple-biomarker approach or require invasive technologies like a surgically placed implant [25-27]. These technologies may not appeal to individuals who prefer a device that calls for help as opposed to delivering naloxone or those who prefer solutions that are short-acting (eg, removable). To expand the pool of possible solutions and to address these limitations, we developed a shoulder-mounted, reusable, rechargeable device with a discreet design and simplified user experience. Unlike Halo, our prototype is designed for extended wear, minimal hardware dependence, and greater comfort and concealment, making it more suitable for continuous monitoring. While other technologies such as automated naloxone implants are emerging, our focus is on non-invasive, user-centered design for early overdose detection outside clinical environments.

### Objective

While previous studies have established preliminary interest among people who inject drugs after viewing prototypes of conceptual devices or wearing a commercially available biosensor patch [28-30], there remains limited research that incorporates consumer feedback after periods of use to inform product development as new devices are being built and tested for market. This study represents a novel attempt to elicit usability, acceptability, and preference data to inform product design during the early development of a shoulder-mounted sensor designed specifically for overdose events.

## Methods

### Study Setting and Participants

Participants were recruited from a walk-in substance use disorder outpatient clinic located in Camden, New Jersey, between September and December 2023. Eligible participants were English-speaking individuals aged ≥18 years, able to provide informed consent, reporting having a secure place to store an electronic device, and reporting injecting illicit opioids >4 times per day in the past 7 days or taking medication for opioid use disorder (MOUD) and injecting an illicit opioid or stimulant in the past 7 days. Flyers with contact information for the study team were visible at the walk-in clinic. Interested individuals were instructed to contact research staff (via telephone or during onsite recruiting) to be screened for eligibility by completing a brief Qualtrics survey. Research staff also conducted face-to-face recruitment in the waiting room of the clinic weekly, during two 4-hour shifts. Research staff enrolled participants sequentially until thematic saturation was reached, or no new patterns or themes emerged from the data [31].

### Measures and Analyses

#### Participant Characteristics

Brief baseline surveys were programmed into Apple iPad tablets. They captured age, race, and ethnicity (White, Black or African American, Hispanic or Latino, Asian, American Indian or Alaska Native, Native Hawaiian or other Pacific Islander, and other) recategorized as non-Hispanic White versus Black, Indigenous, and people of color, gender (man, woman, and nonbinary), housing status recategorized as homeless (staying in shelter, car, street, or abandoned building) versus housed (apartment or house that is owned or rented by participant, partner, family, or friend), education (less than high school, high school or equivalent, some college), past 3-month frequency of injection drug use recategorized as daily or nondaily, average daily number of drug injection events, weekly polysubstance use (yes or no), number of lifetime opioid overdose events, and receiving a prescription for MOUD in the last 30 days (yes or no). Surveys were interviewer-administered and took an average of 20 minutes to complete.

#### Biosensor Data Collection of Physiological Metrics

**Figure 1.**
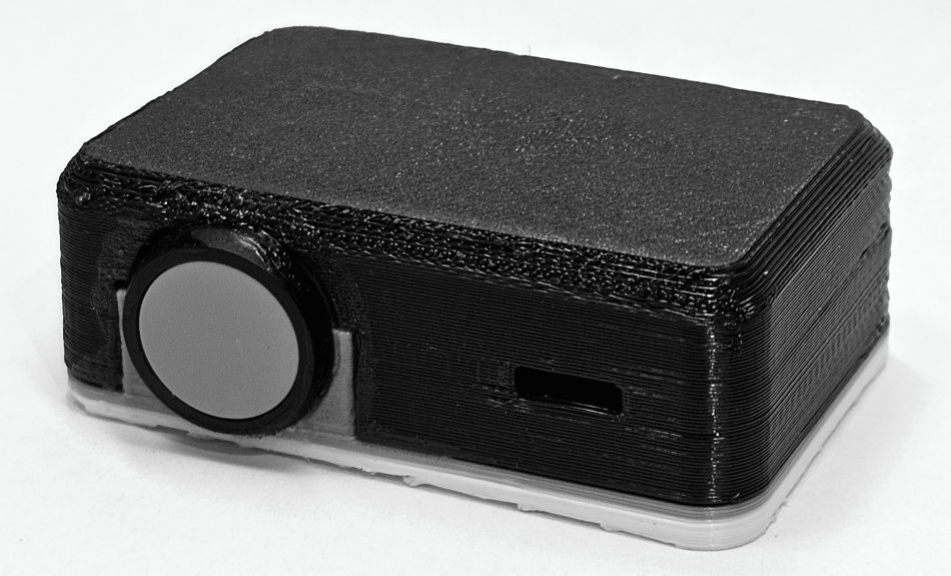
Device prototype. Size: 1.5”x2”x1”.

The biosensor was housed in a 1.5”x2”x1” plastic case (see Figure 1) and was powered by a 2000 mAh lithium-polymer battery designed to last up to 14 days. It was secured to the participant’s deltoid with a medical-grade adhesive patch. The sensor used accelerometry to measure motion and photoplethysmography, a noninvasive technique that uses light shining through the skin to take measurements of heart rate, respiration rate, and oxygen saturation levels. Sensed data were recorded every 25 seconds, data stored locally to the device, and data downloaded upon return of the device. Participants were informed that the devices were prototypes incapable of real-time monitoring and alerts. Participants were instructed to continue regular harm reduction strategies to avoid overdose (eg, taking small amounts of the drug when potency is unknown) or ensure prompt resuscitation (eg, using in the presence of someone equipped with naloxone).

Participants were instructed on proper sensor placement and were assigned a device prototype that they were asked to wear continuously for 7 days, except while showering. Participants practiced placing the device on themselves while being observed by trained interviewers. Once properly secured to the deltoid, baseline heart rate and oxygen saturation measurements were obtained using an FDA-approved pulse oximeter (EMO-80 Pulse oximeter by EMAY). In addition to continuous wear, participants were instructed to depress a button on the prototype (see Figure 1) to indicate when they were about to use drugs to facilitate triangulation of physiological data with self-report.

Before the study described in this manuscript, acceptability studies demonstrated that the form factor, location, and user interface were important to user acceptability [23,28,29]. As such, this prototype was developed and optimized to the acceptability criteria previously published [32]. This noncommercially available prototype was designed and produced by the members of the research team for this and other ongoing projects.

### Qualitative Interviews

After 7 days of wear, participants were instructed to return the device to the research office and were invited to complete a semistructured interview about their experience to inform the product design. Interview questions explored potential harms and benefits, “Thinking back, tell me about any bad [good] things that happened in the past week that you attribute to wearing the device?*”* We also sought to understand the user experience and to identify engineering solutions that could be implemented to improve it, “What it was like to incorporate the device into your daily routine?” with follow-up probes to explore context. “How comfortable is it to wear the device?” with probes to improve comfort and appearance. Interviews were audio-recorded, transcribed verbatim, and checked for accuracy by a member of the study team.

Interviewers (AKD and RL) received training in the qualitative research techniques used in this study from AMR and had opportunities to review a pilot interview and use that to iterate the semistructured guide used in this study before beginning independent data collection. In addition, each interviewer had experience working with women who use drugs before joining the study team. This included roles in emergency and harm reduction settings as well as community-based research studies with people who use drugs. AMR did not participate in data collection but contributed to the analysis, including discussions around discrepancies in the application of codes.

### Analysis

Sociodemographic and behavioral characteristics were summarized using descriptive statistics. Elements of rapid qualitative evaluation were used to examine narrative data [33,34]. Immediately following each interview, research staff completed a structured debriefing report to note observations and key findings relating to domains in the interview guide as well as any new or emerging themes that arose organically during the interview. After interviews were transcribed verbatim, a second team member reviewed the audio file against the verbatim transcript to check for accuracy and simultaneously added their notes on key findings to the debriefing form along with illustrative quotes – data to support the findings. In this way, data collection and analysis were overlapping (debriefing reports) and sequential processes (review of transcripts). Next, data and findings from individual-level debriefing reports were organized in a matrix by interview domain. The matrix was reviewed during group discussions between research team members (AMR, AKD, and RL) who summarized data across all participants by domain, and supporting evidence (eg, quotes) were selected for inclusion in publication.

### Ethical Considerations

The study protocol was approved by the Drexel University and Cooper Hospital institutional review boards (IRB protocol no 2206009308). All participants provided written informed consent and were eligible to receive a maximum of US $110 as compensation for their participation. This was disbursed as US $65 for completing the 7-day study, US $20 for returning the device, and US $25 for participating in the qualitative interview. All participant data were anonymized and labeled only with a unique study identification number. All quotations are presented with participants’ self-selected pseudonyms.

## Results

### Sample Characteristics and Protocol Engagement

Participants (n=23) were primarily white, non-Hispanic (15, 65%), men (14, 61%) with a median age of 41 (IQR 34-46) years, most of whom were unhoused (13, 57%) at the time of enrollment. Most (17, 74%) reported receiving a prescription for MOUD in the past 30 days, and the remainder were attending the clinic for a buprenorphine induction. In the 90 days before enrollment, more than half of participants (13, 57%) reported co-occurring substance use and intravenous drug use an average of 5 (IQR 3.0-8.5) times daily. Most (19, 83%) had experienced an opioid overdose, with a median of 2 (IQR 1-3) lifetime overdose events, and all had witnessed an overdose. Most participants (n=16, 70%) returned their prototype and completed an exit interview. Demographic and behavioral characteristics of participants at baseline are displayed in Table 1.

**Table 1.** Participant characteristics.

Characteristic	All enrolled participants (n=23)	Interview sample (n=16)
Age (years), median (IQR)	41 (34‐46)	41 (36‐44)
Gender, n (%)		
Men	14 (61)	11 (69)
Women	9 (39)	5 (31)
Race and ethnicity, n (%)		
Non-Hispanic White	15 (65)	10 (63)
BIPOC^a^	7 (30)	6 (38)
Education, n (%)		
Less than high school	2 (9)	1 (6)
High school or equivalent	13 (57)	8 (50)
Some college or trade school	8 (35)	7 (44)
Homeless, n (%)	13 (57)	6 (38)
Daily injection drug use^b^, n (%)	17 (74)	12 (75)
Average injection events per day^b^, (n, IQR)	5 (3-9)	5 (3-9)
Weekly polysubstance use^b^, n (%)	13 (57)	9 (56)
Opioid overdoses		
Opioid overdose (ever), n (%)	19 (83)	15 (94)
Median number of overdoses (IQR)	2 (1-3)	3 (2-5)
MOUD^c^ prescription past 30 days, n (%)	17 (74)	11 (69)

a BIPOC includes Black or African American, Hispanic or Latino, Asian, American Indian or Alaska Native, Native Hawaiian or other Pacific Islander, and other.

b In the past 3 months.

c MOUD: medication for opioid use disorder.

### Key Considerations Impacting Acceptability

From the interviews, we identified 4 key themes: perceived threat of overdose, perceived benefits of wearable sensors, perceived risks of wearable biosensors, and design considerations for acceptability. Each is described in the following sections. All quotations are presented with participants’ self-selected pseudonyms.

### Perceived Threat of Overdose

Overdose was commonly reported as a concern and traumatic experience. The highly variable local drug supply was considered a serious threat to participant safety. Because of their personal experiences and perceived susceptibility to overdose, most participants were open to the concept of a wearable device as a new prevention strategy.

*I used Percocet and stuff for years and never overdosed. Then, five times in the first two months with the fentanyl because you just can’t judge, and the strength’s so different…It varies from bag to bag, even parts of bags. I’ve known two people, one OD’d off a bag and one didn’t, so. People don’t know what [drugs] they’re doing... I don’t want to die, just because I use drugs. I deserve -- nobody deserves to OD and die by themself...I’m in recovery. But you never know. You still wear your seat belt even though you know you might not get in an accident.*

[Aristone, age 41, reports daily injection drug use and current homelessness]

### Perceived Benefits of Wearable Biosensors

Perceived benefits of a wearable sensor were both physical (increased likelihood of receiving lifesaving measures in time) and psychological (increasing awareness via biofeedback of the physiological effect of use).

*…And after a while, it might help people get off a drug. That device is good because it’ll help a person slow down with using, and it gives some structure to you inside the drug game also.*

[Michael, age 56, reports daily injection drug use, weekly polysubstance use, and current housing]

Without prompting, participants discussed specific situations when a wearable sensor would be useful, such as when individuals use alone or in spaces where drug use tends not to be disclosed, either because it is prohibited such as their place of employment or in living situations where roommates or family are not aware of their drug use. Preferred options for overdose response were those with the shortest time between overdose and naloxone administration, and those with the highest likelihood of response.

*God forbid, if I’m at work on lunch break, in the bathroom and I shoot up, people ain’t going to come knocking on that bathroom door [until] maybe 20-something minutes later...otherwise, you’re just going to be sitting there and end up dead.*

[Bill, age 51, reports weekly injection drug use, weekly polysubstance use, and current housing]

### Perceived Risks of Wearable Biosensors

While no participant experienced an adverse event, potential social and legal risks were noted. Social risks include potentially dangerous interactions, uncomfortable conversations, and experiencing stigma. Most participants were comfortable disclosing their use with peers but were concerned about situations in which the device’s visibility or its data could be used against them.

*It’s a pain in the ass because people were asking all kind of questions, ‘What is that? Are you a cop?’... Oh, oh, oh, the drug dealers, yeah, when I go to cop on a block, and they see it [it’s] very uncomfortable*.

[Bob, age 50, reports daily injection drug use, daily polysubstance use, and current homelessness]

Concerns about law enforcement were raised in the context of legal risks and surveillance of illicit activities. Many participants voiced strong opinions about law enforcement’s role in overdose response and therefore their role in the device’s functionality.

*I just don’t want the police involved. That would be the only thing. I don’t care, call 911, they just treat you. But if you can get in trouble for it, I think that’s when people hesitate because usually they have [drugs] on them, or they’re afraid of the police getting called.*

[Aristone, age 41, reports daily injection drug use and current homelessness]

However, some participants perceived benefits to outweigh potential risks. For example, we heard,

*… when I was on parole or something like that, some people won’t [use the device] only for legal situations and 911 getting called. That’s the thing. People don’t want to have run-ins with the law. But at the end of the day, if that’s going to save your life then, hey, save it*.

[Amber, age 36, reports weekly injection drug use and current homelessness]

### Design Considerations for Acceptability

Form, function, and accuracy are critical to acceptability. While participants were interested in the device as a concept, the prototype itself had notable design flaws, especially its size, which made it hard to conceal and uncomfortable to sleep in. A discreet, thin, and lightweight device was unanimously endorsed.

*Well, right now, [I wouldn’t want to wear it places] because it’s big and bulky. But if it was a better size and less noticeable, absolutely. . . I think it’s uncomfortable being around other people and them not knowing what’s it’s for.*

[Eric, age 37, reports weekly injection drug use and current homelessness]

*Not that I’m ashamed of it. I just wouldn’t want to explain it to everybody every five minutes.*

[Adam, age 41, reports weekly injection drug use and current housing]

Participants were most interested in a device with customizable responses based on distinct user preferences. A lack of trust in the device’s technological ability to correctly identify an overdose was a common response; many were highly concerned about false positives and receiving an unwarranted intervention, especially one that might involve police.

*You don’t wanna automatically get stuck with the paramedics and cops, just because it was a false, you know? That machine doesn’t know for sure… I wouldn’t trust [an auto-injector]. In a perfect world, that’d be great. But it’s not a perfect world.*

[Oscar, age 39, reports daily injection drug use and current homelessness]

Thus, less invasive response options such as an alarm were preferred over an auto-injection of naloxone, which had the potential for introducing acute precipitated withdrawal.

*I think it would be cool, though, if you had your choice to do it [call 911 or an emergency contact], or depending on that specific person, what they would want done if something like that happened to them.*

[Madonna, age 27, reports daily injection drug use, daily polysubstance use, and current housing]

However, some participants were less concerned about precipitated withdrawal.

*If something’s wrong I guess it really doesn’t matter who comes to your help right? […] Because if something’s wrong and you [receive naloxone] – being sick’s my last worry. That’s [fixable] and your life’s not. I’d feel safer for sure.*

[Sarah, age 34, reports daily injection drug use, and current housing]

## Discussion

### Main Results

This research provides proof of concept for a shoulder-mounted wearable sensor for overdose detection and adds to the growing literature on the acceptability of electronic harm reduction interventions [35]. In this sample, most participants viewed the unpredictable illicit drug market as their greatest risk factor for overdose, likening the uncertainty of what adulterants their bag might contain to a game of “Russian Roulette.” Most had experienced an opioid overdose, and all had witnessed an overdose event. Considering this vulnerability, participants collectively endorsed the importance of wearable overdose detection and response devices as a novel harm reduction tool.

While participants in this pilot noted multiple challenges with the prototype including its bulky size and short battery life, they generally found shoulder placement acceptable. Participants expressed a strong preference for smaller devices that can be worn for longer periods and strategically placed where they will not be identified by others, especially those who might suspect the device is being worn for police surveillance. These findings align with our previous research [23,28] and a recent qualitative study conducted in London, United Kingdom, by Tas et al [29], which used interviews and focus groups to identify factors that might facilitate or hinder wearable device use among individuals taking MOUD. In that sample, drivers of acceptability also included device conspicuousness, the importance of extended battery life, and aesthetics with a preference for a device that could be multifunction [29]. Chapman et al [30] reported that people who inject drugs in Worcester, Massachusetts, who wore a wrist-based sensor expressed similar preferences with size, simplicity of use, comfort, and pleasing aesthetics emerging as important.

When asked to gauge the acceptability of a set of real-time interventions that could be triggered by a next-generation device, we did not identify a single “best fit” solution for the sample. Based on varying preferences in the sample, what emerged is the importance of customizable solutions that can be tailored to fit individual preferences, social contexts, or the environment. Participants expressed a preference for interventions that would alert first responders over automatic biomedical solutions, such as naloxone injection. Most participants had experienced painful precipitated withdrawal after being revived with naloxone and emphasized the critical importance of developing systems that can accurately distinguish between higher-level sedation and overdose, as pointed out in previous studies [23,28,29]. In our study and others, fears of inaccuracy and false alarms emerged as key considerations [29]. Although the impact of tailored response options on device uptake and prolonged use has not yet been studied in the novel overdose prevention technology field, it has been assessed in the biopsychosocial literature, with studies showing that allowing participants to choose from an array of treatment options results in higher uptake and retention over time [36]. While not a part of this study, we intend to assess the impact of choice and customizable response options in future research. Our working hypothesis is that allowing participants to choose may increase engagement and assuage concerns about false positives by allowing participants to opt out of any response considered too risky.

Like Wagner et al [37], we found most participants were wary of any intervention that might trigger a response by law enforcement and lead to their arrest. These concerns echo those in Tracy et al [38], who reported that fear of legal repercussions was associated with delaying or not calling emergency medical services among people who use drugs in New York City who witnessed an overdose event. While no legal or social risks were identified in our sample, they were broadly mentioned as concerns for ongoing wear.

Beyond its potential as a lifesaving device, participants also perceived psychological benefits associated with device wear, particularly its ability to increase reflexivity about drug use patterns. Awareness was considered a potential catalyst for taking steps to reduce use, an important outcome in a group accessing medications for opioid use disorder. Other studies have reported similar results about the potential benefit of using mobile technology to monitor high-volume behaviors (ie, smoking or illicit drug use) [39].

### Limitations

There are several limitations to our data; most notably, our small sample includes individuals who were accessing treatment for opioid use disorder in a single city. This prohibits us from extrapolating findings to other settings or individuals who may benefit from such a device, including those who have not engaged with substance use disorder treatment. Nonetheless, data collection continued until thematic saturation was reached, which is the gold standard for deciding whether to recruit more participants into qualitative research [31]. While participant attrition was 30%, this is a common threshold for technology-based, longitudinal research involving people who inject drugs, who face individual (eg, addiction severity) and structural barriers (eg, homelessness and phone access) to remain engaged in research [40,41]. In this study, participants who were homeless were less likely to complete study activities than those who were housed despite otherwise having similar demographic and behavioral characteristics to the overall sample.

Moreover, the sample was primarily comprised of middle-aged non-Hispanic White men, similar to other studies based in outpatient treatment settings and reflective of the dominant demographic accessing treatment for opioid use disorder [29,42]. This limits our ability to comment on specific age, gender, or cultural impacts on acceptability. We plan to oversample these populations in future studies with larger samples. Further, participants were only asked to wear the prototype for a week, and issues relating to battery life were expressed. This makes it difficult to ascertain proper sensor placement and wear, or long-term acceptability of a device. To understand adoption and use over time, we plan to conduct larger-scale studies of longer duration as part of our ongoing development and testing activities.

### Conclusions

Despite extensive efforts to expand take-home and community naloxone distribution, overdose mortality remains unacceptably high, indicating that additional interventions are necessary to combat this global crisis [43]. The addition of alpha-adrenergic agonists into the opioid drug supply has further complicated overdose assessment and response [44-46]. Technological solutions such as wearable sensors have emerged as a promising approach, particularly because they could facilitate more complex overdose responses, as well as address overdoses that occur during solitary use when bystanders are unavailable to administer life-saving interventions. This research provides valuable insights into factors that may influence the adoption of wearable overdose detection and response technology and highlights key areas for technological and design improvements.

Importantly, the prototype used in this study and the data it collected was not intended to change clinical decision making by the treatment team, nor behavior by the patients. That said, given the results of this study, there are several clear steps that need to be taken before it could be safely used in real-world situations. First, the technical hurdle of reliable demonstration of accuracy of physiological parameter monitoring would need to be passed, using a gold standard for pulse oximetry such as arterial blood gas analysis. We would also need to address issues of battery life, aesthetics, and discreet placement. Secondly, an analysis of the ethical risks of such a device, in terms of modifying patient behavior in a more hazardous way, would need to be rigorously performed. However, research across settings suggests that improving access to overdose reversal agents (ie, take-home naloxone) does not result in risk compensation by people who inject drugs, meaning they do not assume additional risk, like using larger volumes of illicit opioids or using more frequently, because their concerns about overdose are diminished [47,48]. Finally, a clear blueprint for implementation of the physiological monitoring and a strategy for ethical implementation would have to be presented to the federal regulatory authority for clearance. Based on the results of this study and others, the device is being moved forward along this pathway. As we navigate this process, we continue to conduct user experience research with the goal of meeting end user needs.

## Abbreviations

FDA: Food and Drug Administration
MOUD: medication for opioid use disorder
